# The prognostic predictive value of systemic immune index and systemic inflammatory response index in nasopharyngeal carcinoma: A systematic review and meta-analysis

**DOI:** 10.3389/fonc.2023.1006233

**Published:** 2023-02-03

**Authors:** Li Wang, Xianfei Qin, Yu Zhang, Shouyu Xue, Xicheng Song

**Affiliations:** ^1^ Otorhinolaryngology Head and Neck Surgery, Yantai Yuhuangding Hospital, Yantai Shandong, China; ^2^ School of Clinical Medicine, Binzhou Medical University, Yantai, China

**Keywords:** systemic immune index, systemic inflammatory response index, nasopharyngeal carcinoma, meta-analysis, prognosis

## Abstract

**Objective:**

To study the predictive value of systemic immune index (SII) and systemic inflammatory response index (SIRI) in the prognosis of patients with nasopharyngeal carcinoma.

**Methods:**

Two researchers independently searched PubMed, Cochrane, Embase, and Web of Science databases (until March 18, 2022) for all studies on SII, SIRI, and prognosis in patients with nasopharyngeal carcinoma. Quality assessment of included studies was assessed using the Newcastle-Ottawa Scale (NOS). In addition, a bivariate mixed-effects model was used to explore predictive value.

**Results:**

A total of 9 studies that satisfied the requirements were included, involving, 3187 patients with nasopharyngeal carcinoma. The results of the meta-analysis showed that SII could be an independent predictor of OS (HR=1.78, 95%CI [1.44-2.20], Z=5.28, P<0.05), and SII could also be an independent predictor of PFS (HR=1.66, 95%CI [1.36-2.03], Z=4.94, P<0.05). In addition, SIRI could also serve as an independent predictor of OS (HR=2.88, 95%CI [1.97-4.19], Z=5.51, P<0.05). The ROC area was 0.63, the sensitivity was 0.68 (95%CI [0.55-0.78]), and the specificity was 0.55 (95%CI [0.47-0.62]), all of which indicated that SII had a certain predictive value for OS.

**Conclusion:**

SII and SIRI can be used as independent predictors to predict the prognosis and survival status of patients with nasopharyngeal carcinoma and have certain predictive accuracy. Therefore, SII and SIRI should be considered in studies that update survival risk assessment systems.

**Systematic Review Registration:**

https://www.ytyhdyy.com/, identifier PROSPERO (CRD42022319678).

## 1 Introduction

Nasopharyngeal carcinoma (NPC) is a malignant tumor that occurs in the epithelium of the nasopharyngeal mucosa, and its incidence is the first of the malignant tumors of the ear, nose, and throat (1). According to the International Agency for Research on Cancer in, 2018, there were about 129 079 new cases of NPC and 72,987 deaths worldwide each year (2). About 80% of NPCs in the world occur in China (3). China’s Guangdong Province and Hong Kong Special Administrative Region are the high-incidence areas (4). At present, radiotherapy is the primary treatment method for NPC. With the promotion of comprehensive treatment, the therapeutic effect and prognosis of NPC patients have been significantly improved. However, local recurrence or metastasis occurs in 10% to 20% of patients (5). Thus, recurrence and distant metastasis are the main reasons for treatment failure. This is mainly due to the occult location of NPC and the difficulty distinguishing symptoms from other benign diseases; most patients with NPC are diagnosed in the middle and late stages, with cervical lymph node and/or distant metastasis (6). Therefore, it is significant to explore early biomarkers for predicting the prognosis of NPC and intervening in the treatment measures for patients with high-risk factors to improve the overall prognosis of NPC.

Currently, the most widely used and mature diagnostic and prognostic markers are EBV-related markers. This is because non-keratinizing NPC is the main type of NPC, and Epstein-Barr virus (EBV) infection is the main pathogenic factor of non-keratinizing NPC (6). Among them, plasma EBV DNA detection is currently the most widely used clinical method for detecting NPC markers. In addition, researchers have also carried out certain research and exploration on a variety of Micro RNAs (7), multi-molecular markers (8, 9), and methylation markers (10, 11). However, these detection methods are relatively complicated, the detection cost is high, and the results vary greatly, limiting their promotion and practical application. Therefore, researchers continue to develop more economical, convenient, and stable markers to enhance the prognosis of NPC patients.

Many recent studies have found that inflammation is crucial in cancer occurrence, development, and prognosis. Studies have confirmed that chronic inflammation is inseparable from various stages of tumorigenesis, proliferation, infiltration, metastasis, and apoptosis (12). Cancer-related inflammation has been listed as one of the ten characteristics of cancer (13). Relevant studies have proved that systemic inflammatory response indicators can well evaluate and predict tumor development and prognosis. The commonly used systemic inflammatory response indicators include platelet/lymphocyte ratio (PLR), neutrophil/lymphocyte ratio (NLR), monocyte/lymphocyte ratio (Monocyte-lymphocyte ratio) ratio, MLR), C-reactive protein to albumin ratio (CRP/Alb), etc. (14–16).

Systemic immune-inflammation index (SII) and systemic inflammatory response index (SIRI) are new indicators that reflect human inflammation and have become a research hotspot in recent years. SII is defined as (platelet count × neutrophil count)/lymphocyte count, which can better reflect the potential indicators of inflammation and immune balance in the host (17). The systemic inflammatory response index (SIRI), defined as (neutrophils × monocytes)/lymphocytes, is a good indicator of cancer-related inflammatory responses (18). Although many studies have affirmed the value of SII and SIRI in predicting the prognosis of NPC, some studies have reached the opposite conclusion (19). Elevated SII and SIRI, as independent predictors of relatively poor prognosis in patients with NPC, are more accurate in prognostic prediction than other systemic inflammation markers such as NLR, PLR, and LMR. Therefore, to more comprehensively assess the prognostic value of SII and SIRI in NPC, we conducted a new meta-analysis to investigate the association between SII/SIRI and the prognosis of NPC, providing a strong basis for clinicians to select individualized treatment regimens.

## 2 Methods

Our project is carried out strictly following the PRISMA2020 guidelines, and the registration is completed on the PROSPERO platform after the search. (CRD42022319678)

### 2.1 Retrieval strategy

We performed a comprehensive literature search in the PubMed, Cochrane, Embase, and Web of Science databases using the following keywords to identify all relevant studies on the prognostic value of the SIRI and SII in NPC patients published up to March 18, 2022: (“Nasopharyngeal Carcinoma” OR “Carcinoma, Nasopharyngeal” OR “Carcinomas, Nasopharyngeal” OR “Nasopharyngeal Carcinomas” AND (“Systemic immune index” OR “systemic immune-inflammation index” OR “SII” OR “Systemic inflammatory response index” OR “systemic inflammation response index” OR “SIRI”). This study is limited to the articles published in English. The references and citations of the retrieved publications were also examined to identify other relevant studies.

### 2.2 Inclusion and exclusion criteria

The inclusion criteria were as follows:

1. The subjects of the study were patients with pathologically diagnosed NPC;
2. Studies investigating the association of SIRI or SII with overall survival (OS), progression-free survival (PFS), disease-free survival (DFS), recurrence-free survival (RFS), or clinicopathological characteristics;
3. Studies report cut-off values for SIRI and SII and provide sufficient information to directly or indirectly estimate hazard ratios (HR) and 95% confidence intervals (CI).

The exclusion criteria are as follows:

1. Reviews, case reports, meta-analyses, or conference abstracts;
2. Vitro experiments, animal experiments;
3. Duplicate literature;
4. Studies with full text not available or with missing data (insufficient literature data to calculate HR and 95% CI);
5. Literature in languages other than English.
6. The same source of cases in different studies.

Furthermore, to avoid duplication, only the most informative studies were included when multiple studies were based on the same dataset.

### 2.3 Literature screening and data extraction

Two researchers independently extracted, organized, and analyzed the data of the eligible literature that were finally included. The extracted information consists of the first author’s name, publication year, country, study type, exposure factors, prognostic indicators, number of cases, gender, age, treatment information, follow-up time, the cut-off value of exposure factors, and outcome indicators.

### 2.4 Quality assessment

Two researchers used the Newcastle-Ottawa Scale (NOS) to evaluate the literature quality and cross-check after completion. A third investigator is invited to assist in adjudication if there is a dispute. Among them, the NOS scale includes three aspects and a total of 8 items: 4 items for research object selection, 1 item for comparability between groups, and 3 items for outcome measurement; except for comparability, which can be scored up to 2 points, other the maximum score for an item is 1 point, and the score ranges from 0 to 9 points. The higher the overall score, the higher the quality of the study. The total score is 9 out of 9, with a score of 7-9 considered high quality. If literature contains multiple cohorts, score them separately.

### 2.5 Outcomes

The primary outcome measure in this systematic review was the hazard radio(HR) reflecting the multivariate cox regression between SIRI/SII and the prognosis of NPC. Furthermore, this study discussed the predictive value of SIRI and SII for the prognosis of NPC. Hence, the outcome measures also included sensitivity, specificity, and summary receiver operating characteristic curve (SROC).

### 2.6 Data analysis

This Meta-analysis uses Stata15.0 (StataCorp LLC, College Station, TX) for data analysis. The model selection is based on the heterogeneity index (I2). When I^2^>50%, a random effect model was used; I^2^<50%, a fixed-effects model, was used. When too much heterogeneity existed, sensitivity analysis and subgroup analysis were used to explore the source of heterogeneity. Funnel plots were used to reflect publication bias within each index and among studies intuitively, and Egger’s test was used for statistical testing of publication bias. When there was publication bias, the cut-and-fill method was used to analyze the impact of publication bias on the results of the Meta-analysis.

We used a bivariate mixed-effects model to explore the predictive value of prognosis. We calculated the point estimates of sensitivity, specificity, positive likelihood ratio, negative likelihood ratio, and diagnostic odds ratio for each group and their corresponding 95% CIs. Subject operating characteristics (SROC) were calculated, and AUC and its 95% CI were calculated. Deek’s funnel plot determined publication bias. When P<0.05, the difference was statistically significant.

## 3 Results

### 3.1 Literature search

**Figure 1:**
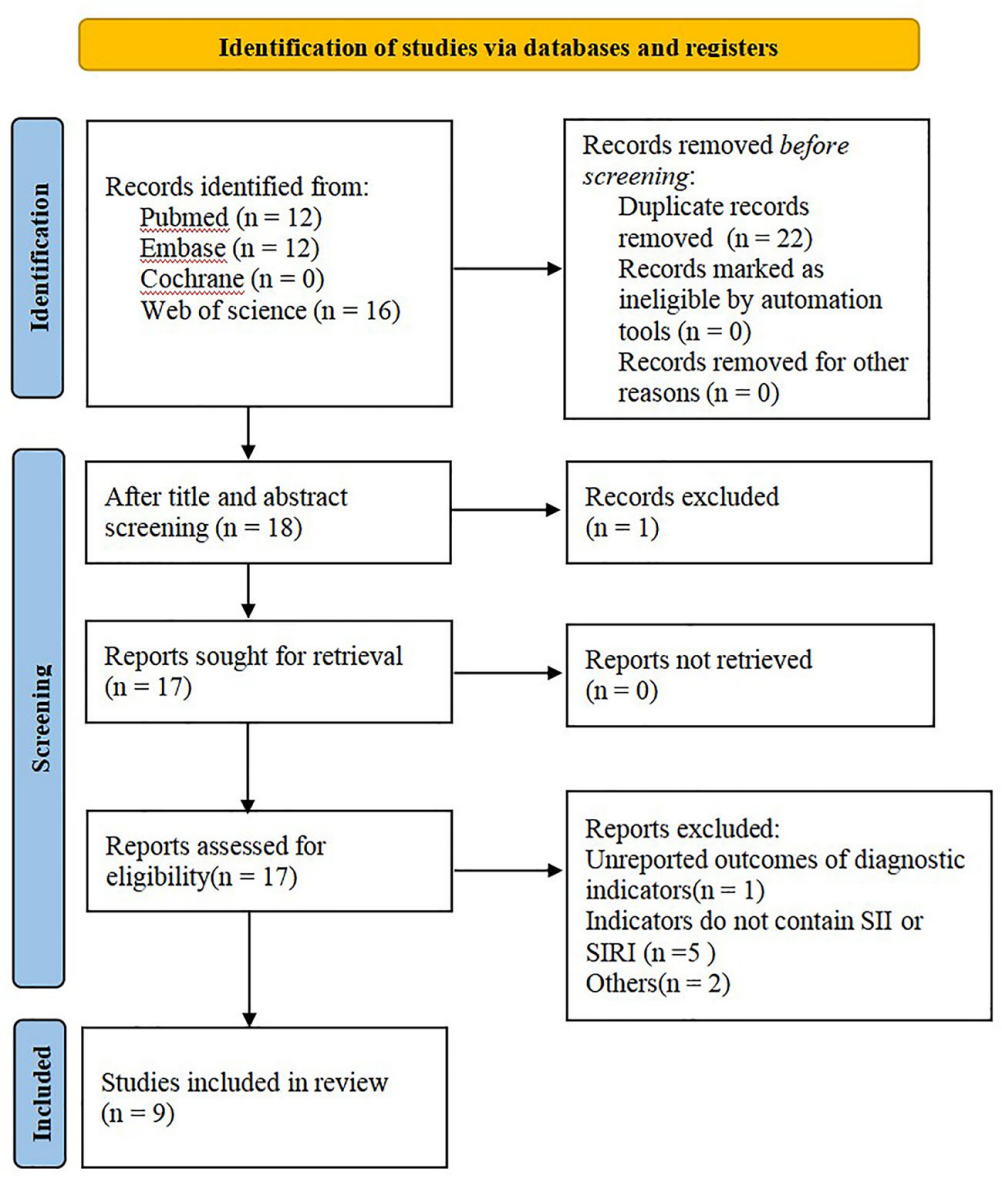
PRISMA flowchart of literature search and selection criteria.

A total of 40 articles were retrieved from the aforesaid databases, and 22 duplicate studies and 1 review were excluded through topic screening by two researchers. After reading the full text, we excluded 1 study that had the same source of cases and 7 articles that did not meet the inclusion criteria or had no SII and SIRI indexes. Nine studies were finally included in this meta-analysis (**Figure 1**).

### 3.2 Eligible studies and the characteristics and quality evaluation

From 9 (19–27) retrospective studies published from, 2017 to, 2021, 3187 cases were included in our analysis. Of these studies, 8 included SII, 5 included SIRI, 9 reported OS, six also reported PFS, and only three reported DMS. All studies were conducted in China. All studies’ treatment methods were conservative, including radiotherapy, induction chemotherapy, and concurrent chemoradiotherapy. In one report, only a few patients with lung metastases were treated with surgical resection. All studies performed univariate and multivariate analyses; only one study performed only multivariate analyses. All studies received a score of 7 or 8 according to the NOS, indicating that all studies were of high quality (**Tables 1**, **2**).

**Table 1:** Basic characteristics of studies included into the meta–analysis.

No.	Author	Year	Country	Design	Exposure	Samplsize	Sex(M/F)	Age	Follow up
1	Qian Li (20)	2021	China	cohort	SIRI	342	247/95	49(rang:16~83)	9Y
					SII				
2	Yibiao Chen (19)	2021	China	cohort	SIRI	216	142/74		>5Y
					SII				
3	Ying Xiong (21)	2021	China	cohort	SII	213	155/58	45(rang:16~70)	7Y
4	Yuhua Feng (22)	2020	China	PSM	SIRI	417	321/96	47(rang:14~81)	8Y
					SII				
5	Xiaojiao Zeng (23)	2020	China	cohort	SIRI	559	421/138	51(rang:12~83)	12.6Y
					SII				
6	Cheng Lin (24)	2019	China	PSM	SII	243	184/59	48(rang:17~81)	11.25Y
7	Yuan Chen (25)	2019	China	cohort	SIRI	285	210/75	22~80	5Y
8	Ronald Wihal Oei (26)	2018	China	PSM	SII	585	420/165	49(rang:17~82)	7.2Y
9	Wenjie Jiang (27)	2017	China	PSM	SII	327	243/84	50(rang:20~80)	13.7Y

**Table 2:** Quality evaluation of studies included into the meta–analysis.

First author	Published years	v1	v2	v3	v4	v5	v6	v7	v8	Total score
Qian Li (20)	2021	★	★	★		★★	★		★	7
Yibiao Chen (19)	2021	★	★	★		★★	★		★	7
Ying Xiong (21)	2021	★	★	★		★★	★	★	★	8
Yuhua Feng (22)	2020	★	★	★		★★	★	★	★	8
Xiaojiao Zeng (23)	2020	★	★	★		★★	★		★	7
Cheng Lin (24)	2019	★	★	★		★★	★	★	★	8
Yuan Chen (25)	2019	★	★	★		★★	★	★	★	8
Ronald Wihal Oei (26)	2018	★	★	★		★★	★	★	★	8
Wenjie Jiang (27)	2017	★	★	★		★★	★	★	★	8

### 3.3 Meta-analysis

#### 3.3.1 The relationship between SII, SIRI, and prognosis of NPC

**Figure 2:**
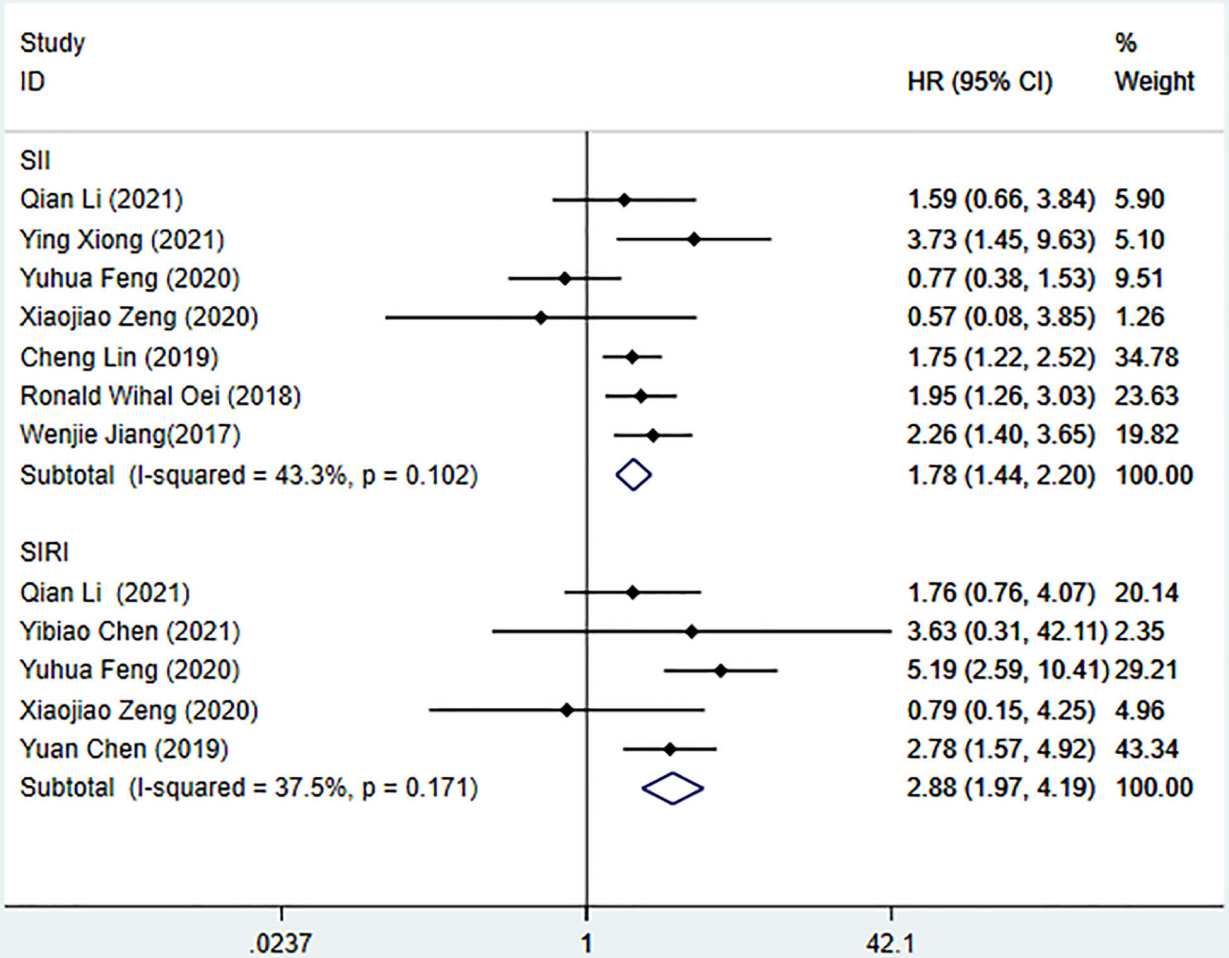
Meta-analysis of the relationship between SII, SIRI and OS in patients with nasopharyngeal carcinoma.

**Figure 3:**
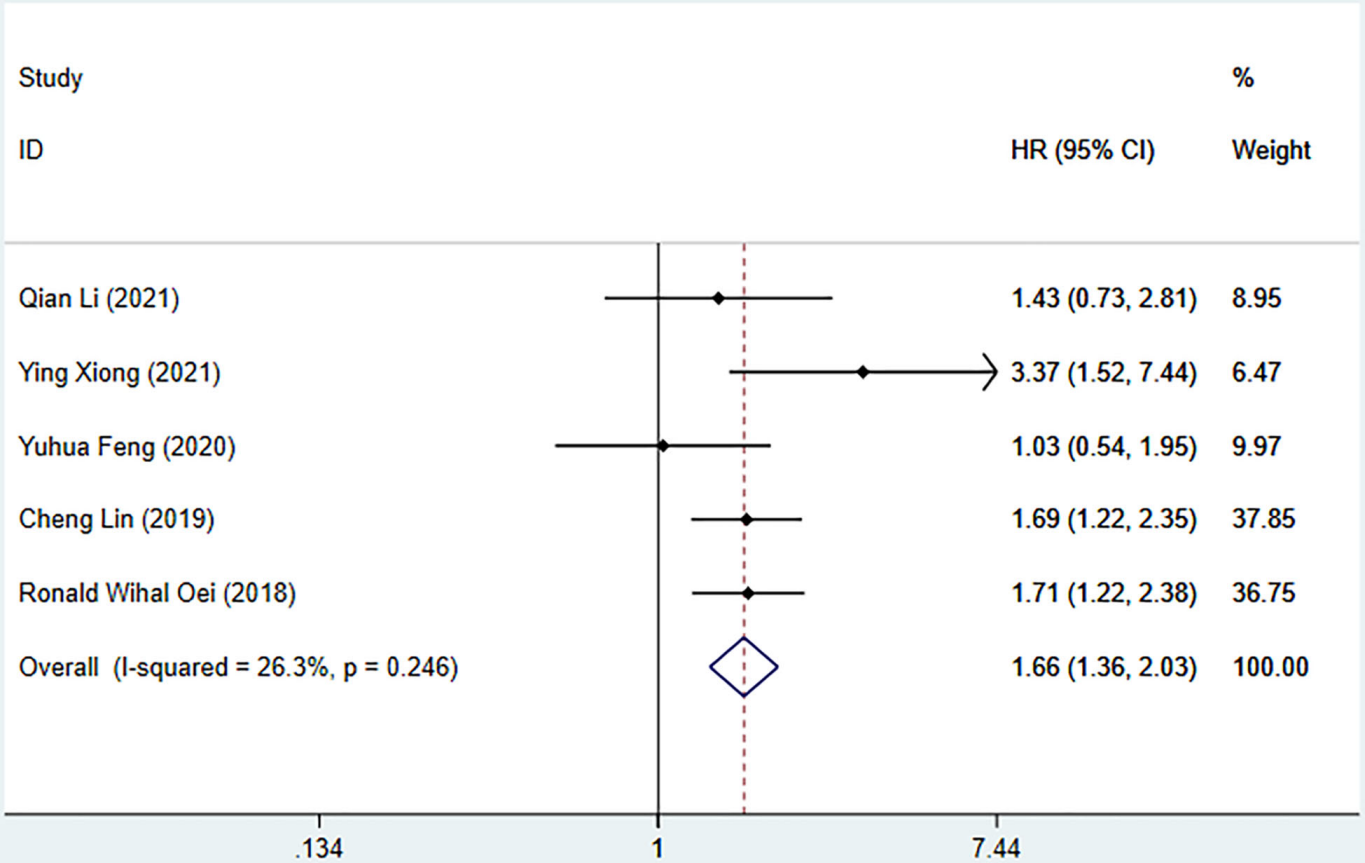
Meta-analysis of the relationship between SII and PFS in patients with nasopharyngeal carcinoma.

Seven studies described the relationship between SII and prognosis in NPC, using a fixed-effects model (I^2 =^ 43.3%, P=0.102) to pool the effect size, the results showed that there was a significant correlation between SII and OS (HR=1.78, 95%CI [1.44-2.20], Z=5.28, P<0.05), and the results of 5 studies showed that there was also a significant correlation between SII and PFS (HR=1.66, 95%CI [1.36-2.03], Z=4.94, P<0.05), indicating that SII was an independent predictor of NPC prognosis. Five studies described the relationship between SIRI and prognosis in NPC. Using a fixed-effect model (I^2 =^ 37.5%, P=0.171) to pool the effect size, the results showed that SIRI was significantly correlated with OS (HR=2.88, 95%CI [1.97-4.19], Z=5.51, P<0.05), indicating that SIRI is an independent predictor of NPC prognosis (**Figures 2**, **3**).

**Figure 4:**
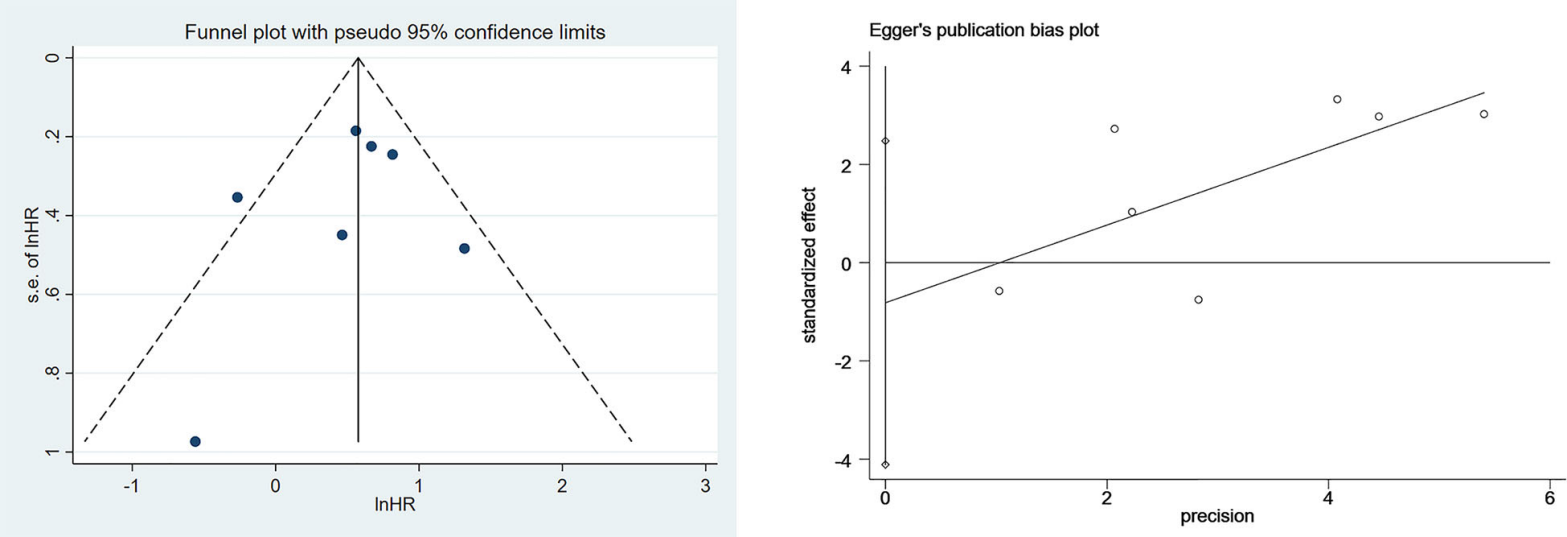
Egger’s test result and funnel plot for the association between SII and OS.

Funnel plots and Egger’s tests were used to analyze publication bias. The funnel plot for the association between SII and OS was basically symmetric, and Egger’s test showed no publication bias (P=0.552) (**Figure 4**). Due to a limited number of studies (n=5) on the associations between SII and PFS as well as SIRI and OS, we did not perform the Egger’s test for these associations.

#### 3.3.2 Predictive value of SII for OS

**Figure 5:**
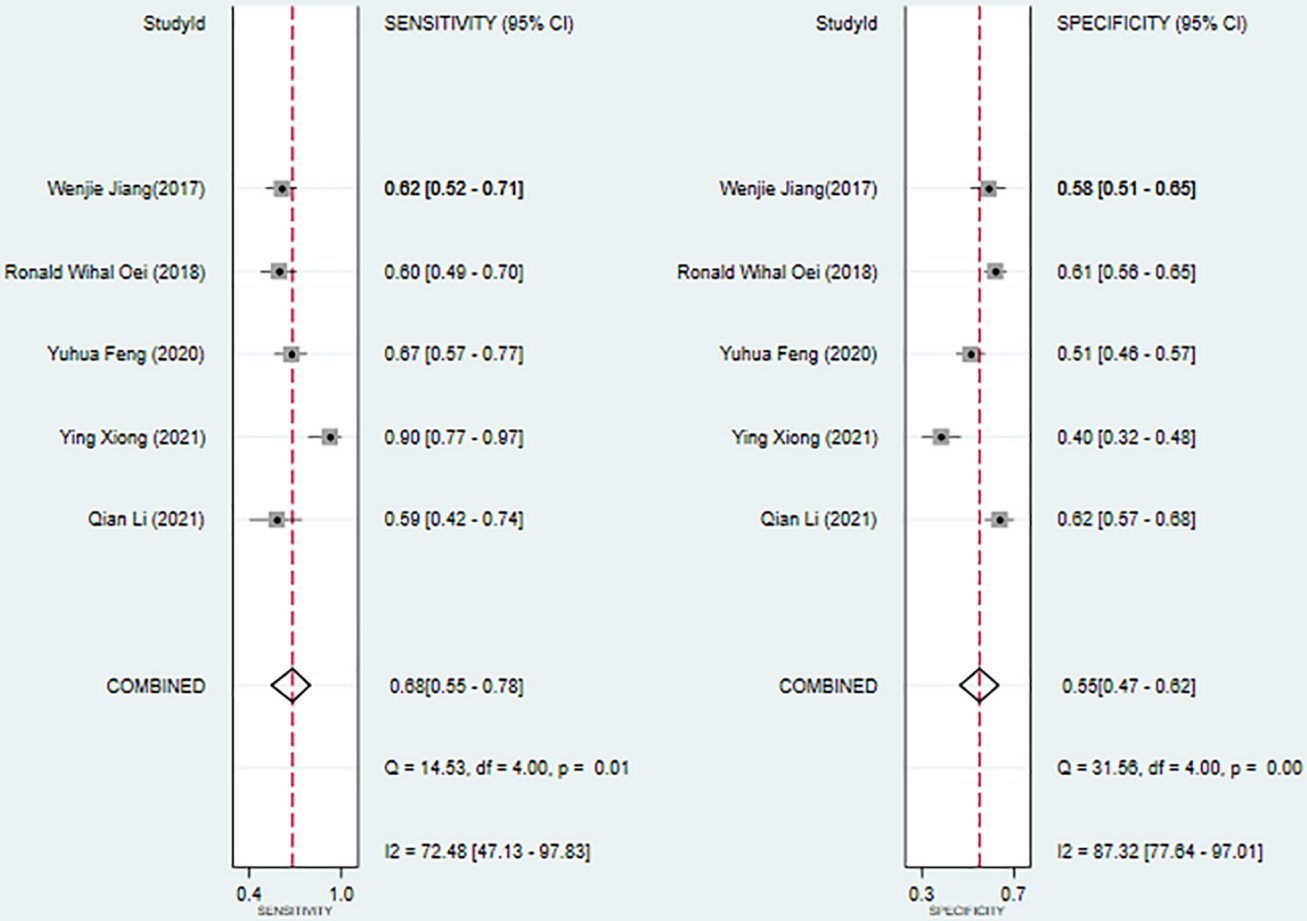
Sensitivity and specificity of SII in predicting OS in nasopharyngeal carcinoma patients.

**Figure 6:**
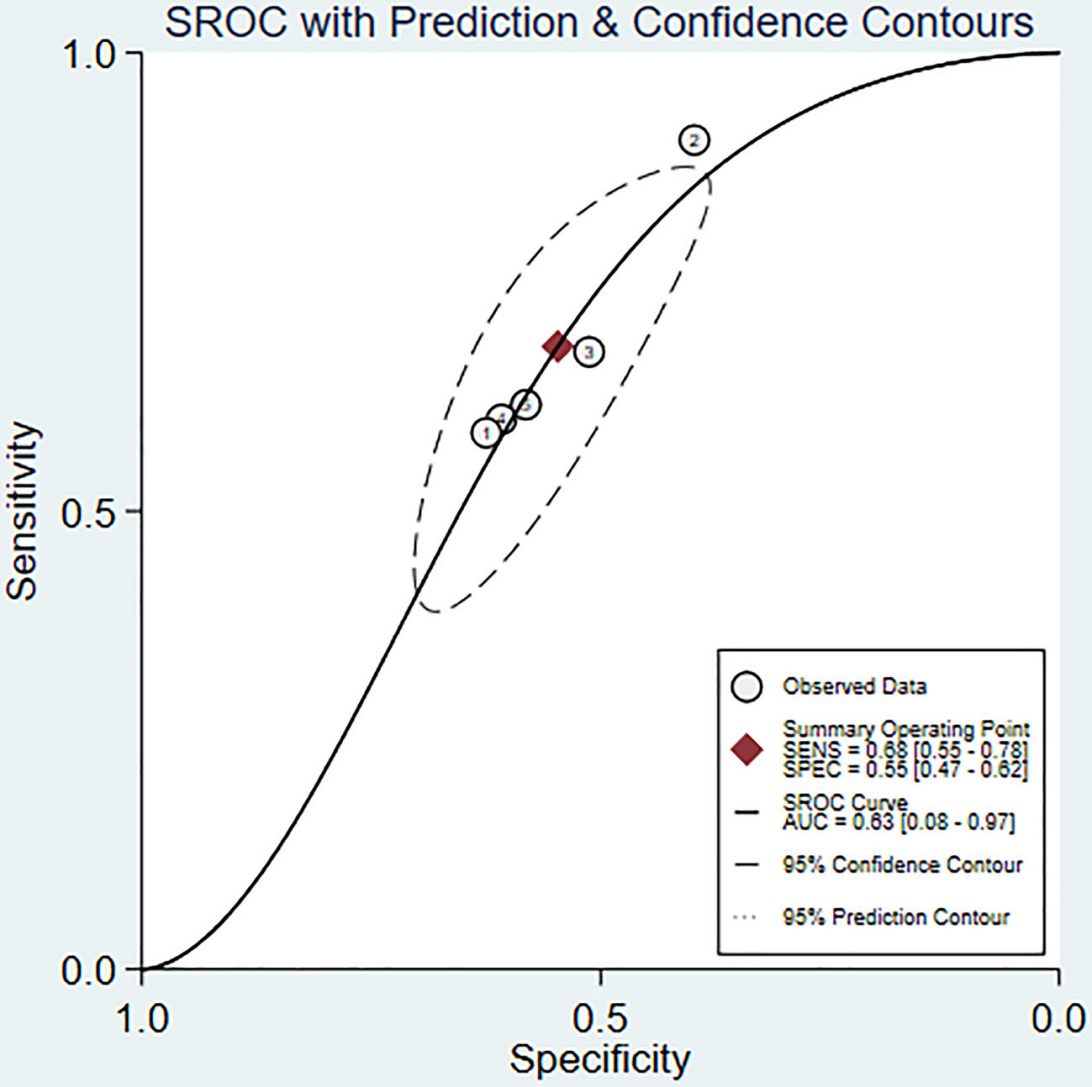
SROC curve of SII predicting OS in nasopharyngeal carcinoma patients.

**Figure 7:**
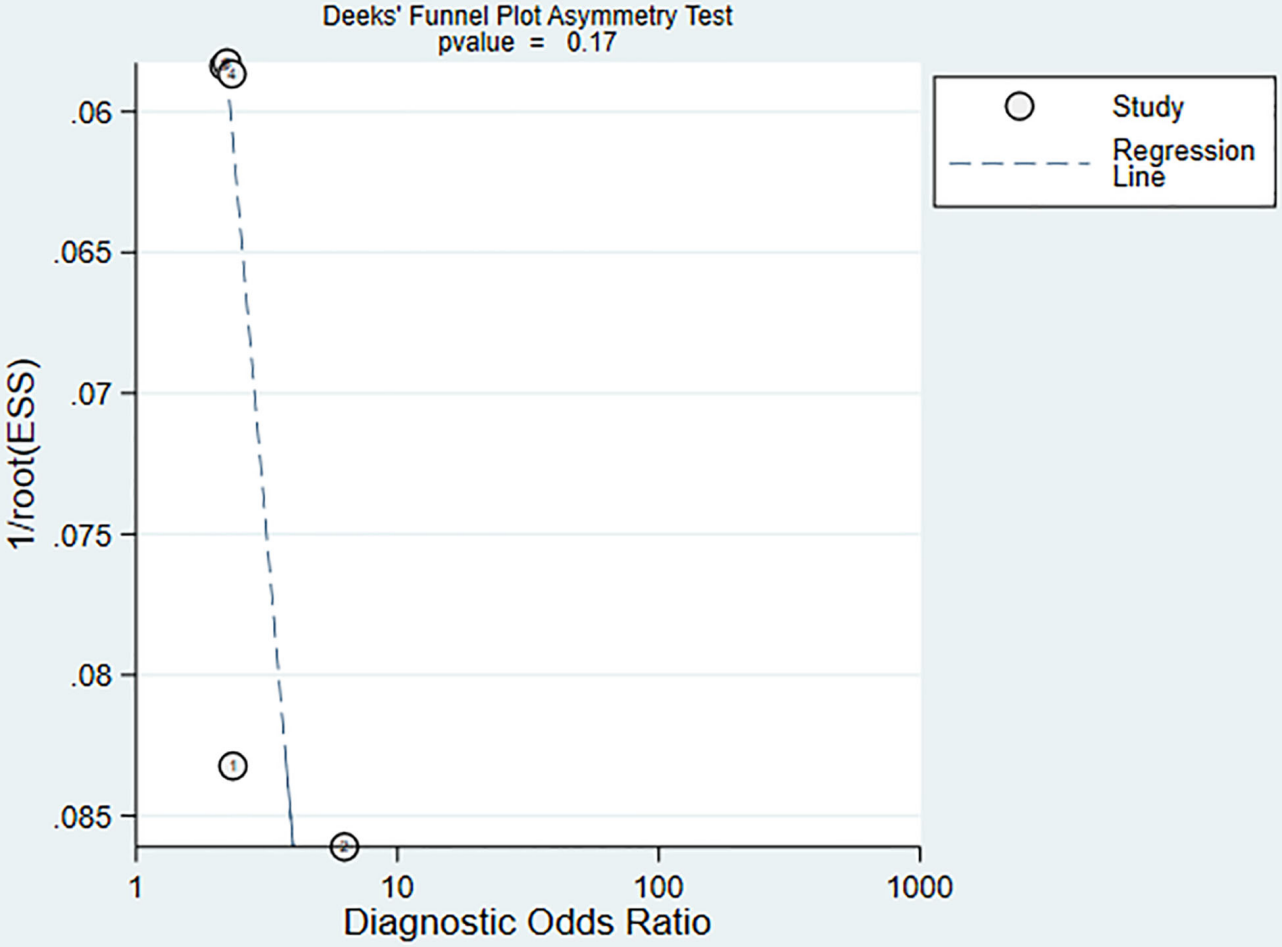
SII predicts OS publication bias in nasopharyngeal carcinoma patients.

We used a bivariate mixed effects model (I^2 =^ 96%) to analyze the predictive value of SII for OS. The analysis showed that the sensitivity was 0.68 (95%CI [0.55-0.78]), and the specificity was 0.55 (95%CI [0.47-0.62]), the positive likelihood ratio (PLR) was 1.5 (95%CI [1.4-1.7]), the negative likelihood ratio (NLR) was 0.59 (95%CI [0.45-0.76]), and the diagnostic odds ratio (DOR) was 3 (95%CI [2-4]), SROC was 0.63 (95%CI [0.58-0.67]). It shows that SII has only a certain predictive value for OS. The included studies may have some publication bias (P=0.17) (**Figures 5**–**7**).

## 4 Discussion

The incidence of NPC is the first among otorhinolaryngology malignant tumors (1), which seriously threatens human life and health. The determinants of early recurrence of NPC remain unknown, so exploring the predictors of the prognosis of NPC has always been the focus of clinical research. Useful predictors can help clinicians adopt effective and individualized therapies for different patients in a timely manner. At the same time, the toxic reaction caused by overtreatment can be avoided.

This meta-analysis included 9 studies with a total of, 3187 patients. The results showed that the levels of SII and SIRI were significantly correlated with OS, and there was also a significant correlation between SII and PFS. The correlation between SIRI and PFS was not calculated because the sample size was too small. The current study also suggests that SII can effectively predict NPC, but more research is needed to develop its predictive value.

Inflammation plays a critical role in the occurrence and development of many malignant tumors (12, 28). Inflammatory markers have been widely used in lung cancer, gastric cancer, colorectal cancer, liver cancer, pancreatic cancer, and other malignant tumors to predict the prognosis of patients because of their cheap detection cost and stable and convenient results. They have become a hot spot in tumor research in recent years. However, studies of inflammatory markers in NPC are relatively few, with mixed conclusions. At present, many prognostic indicators of inflammation have been studied in NPCs, such as neutrophil/lymphocyte ratio (NLR) (29–31), C-reactive protein (CRP) (32, 33), platelet/lymphocyte ratio (PLR) (34, 35), monocyte/lymphocyte ratio (MLR) (36), etc., found that most of the higher prognostic indicators of inflammation were associated with poor prognosis in NPC.

The systemic inflammatory response index (SIRI) is a new index based on immune cells in the human circulatory system (37). It can systematically reflect the complex interactions and potential synergistic effects between neutrophils, monocytes, and lymphocytes in the tumor microenvironment, reflecting the balance between the body’s inflammatory response and immune response state, and can be used to assess the body’s Immune Function. In recent years, a large number of clinical studies have explored the correlation between SIRI and the prognosis of patients with different tumors such as digestion (38–40), respiratory (41, 42), endocrine (43), and urinary (44, 45), and reproduction (46). The results show that SIRI has good predictability for the prognosis of patients with various types of tumors; a higher preoperative SIRI value often indicates a poor prognosis for the patient, so it has a certain guiding role for the clinical treatment of the patient.

The Systemic Immune Inflammation Index (SII) is a newly proposed comprehensive inflammation index. It combines the cell types of platelets, neutrophils, and lymphocytes to comprehensively reflect the host’s immune status and inflammatory response—Independent risk factors for developing solid tumors (47). In recent years, a large number of clinical studies have investigated the correlation between SII and the prognosis of patients with different tumors such as digestion (48), respiratory (49), blood (50), urinary (51), and reproductive (52). It has been investigated in terms of the properties of SII, which shows that compared with NLR, MLR, PLR, etc., SII is more meaningful in predicting the efficacy and prognosis of tumor patients and is an independent prognostic factor for various tumors.

Neutrophils are the most common immune cells in human peripheral blood and can promote tumor angiogenesis (53) Neutrophils can promote tumor invasion and metastasis by secreting cytokines (such as vascular endothelial growth factor (VEGF)), specific proteases (such as matrix metalloproteinases and elastases), and chemokines (54, 55), can also inhibit the antitumor effect of cytotoxic T cells and NK cells (56, 57). Elevated neutrophils can also release a large amount of nitric oxide, arginase, and reactive oxygen species, leading to T cell activation disorder, thereby inhibiting the body’s killing effect on tumor cells and indirectly contributing to tumor progression (12, 58). He et al. (59) reported that neutrophils are an independent biomarker of poor prognosis in NPC patients. Jin et al. (30) found that the increase of neutrophil count before treatment was significantly correlated with the poor prognosis of NPC. 

Lymphocytes are a type of cell population with immune response and regulatory functions. They are also one of the indispensable defense cells of the immune system against tumor cell proliferation. Lymphocytes can be divided into lymphocyte subsets such as T lymphocytes, B lymphocytes, and natural killer (NK) cells. Among them, the role of T lymphocytes is mainly manifested in cellular immunity. When it encounters the stimulation of tumor antigens, It can directly kill target cells and is the front-line fighter of the body’s immune system to play an anti-tumor effect; B lymphocytes are mainly involved in humoral immunity, which can control tumor cells by secreting cytokines such as interferon-gamma and tumor necrosis factor-alpha. NK cells can directly kill malignant tumor cells without antigen stimulation or antibody production. Because of this, they are also called natural killer cells, so the reduction in the number of lymphocytes and Both/or loss of function can impair immune surveillance and defense mechanisms of tumor cells (60). He et al. (59) have discovered that a higher number and percentage of peripheral blood lymphocytes are associated with long-term survival in NPC patients and are independent prognostic factors for NPC patients. Liu et al. (61) have pointed out that treatment-related lymphopenia is a factor for poor prognosis in patients with NPC.

Platelets can promote tumor cell growth, metastasis, and tumor angiogenesis. Studies have found that platelets can promote epithelial-mesenchymal transition (EMT) of circulating tumor cells, increase their motility and apoptosis resistance, and promote tumor cell extravasation (62, 63). Activated platelets form a complex tumor microenvironment that supports tumor cell progression by releasing numerous molecules. Platelets protect circulating tumor cells from damage by NK cells and inhibit NK cell cytotoxicity (64). Liu et al. (65) conducted a meta-analysis on the association between platelet count and the 1-year survival rate of patients with cancer cachexia, and they pointed out that platelet count was negatively correlated with the 1-year OS in patients with cancer cachexia. Xie et al. (66) found that high platelet distribution width (PDW) and platelet count might be prognostic predictors for NPC patients (34). Another study showed that an elevated platelet-to-lymphocyte ratio was associated with poor cancer-specific survival, OS, and distant metastasis-free survival in patients with NPC.

Monocytes can promote tumorigenesis by producing a variety of immunosuppressive, tumor-promoting chemokines/cytokines (67) and further differentiate into tumor-associated macrophages (TAMs). TAMs can induce apoptosis of CD8+ T cells with anticancer activity and promote tumor growth, invasion, and migration (68). In addition, studies have found (69) that the M2 type of TAMs can promote tumor growth, remodel tissue, promote angiogenesis, and inhibit adaptive immunity. Previous studies (70, 71) have revealed that a higher monocyte count is associated with poor prognosis in various types of tumors.

Combined with the above analysis, it is not difficult to understand that the two comprehensive inflammatory indicators, SII and SIRI, can reflect the host immune status and inflammatory response and become the predictors of the occurrence and prognosis of various tumors.

The present study analyzes the role of SII and SIRI in the prognosis of NPC, which is a research focus. Despite the fact that the impact of tumor microenvironment on tumorigenesis, progression, and prognosis is clear, the use of circulatory system tests to understand patients’ immune and inflammatory status and determine their association with the prognosis is controversial and debatable. The present study elucidates the association between SII and the overall prognosis of NPC. SII and SIRI should be considered in the studies on updating the survival risk assessment system. Nonetheless, the association between SIRI and the overall prognosis of NPC remains unknown due to a small number of cases. To confirm their association, more cross-ethnic and multi-center studies are needed.

There are certain advantages to this meta-analysis. First, to the best of our knowledge, this is the first article to study the correlation between SII and SIRI and the prognosis of patients with NPC. Secondly, this study is innovative to a certain extent and has new significance for predicting the prognosis of patients with NPC and subsequent research.

There are still some limitations of this study. First, publication bias was found in the statistical analysis. So we should look at the results of this meta-analysis objectively. Second, the included studies are mainly from China, and high-quality studies from more regions are needed in the future. Third, subgroup analysis was not performed due to limited available data. Fourth, the cut-off values of the SII and SIRI indices selected in the literature included in this study are inconsistent among studies, which may lead to potential selection bias. When the SII and SIRI indices are used in clinical practice, a uniform and fixed cutoff value or reference range is still needed, which requires more multicenter and multisample studies further to determine the optimal cutoff value or reference range.

## 5 Conclusion

SII and SIRI can be used as independent predictors for predicting the prognosis and survival status of patients with NPC and have certain predictive accuracy. Therefore, SII and SIRI should be considered in studies that update survival risk assessment systems.

## Data availability statement

The original contributions presented in the study are included in the article/supplementary material. Further inquiries can be directed to the corresponding authors.

## Author contributions

LW and XQ contributed equally to this work. LW and XQ: Conceptualization, Methodology, Software, Writing- Original draft, Data curation, Visualization were performed; YZ and SX: Investigation, Writing - Original Draft, Writing - Reviewing and Editing were performed; XS: Conceptualization, Supervision, Project administration. All authors contributed to the article and approved the submitted version.
